# A Three-Stage Cellular Automata Model of Complex Large Roundabout Traffic Flow, with a Flow-Efficiency- and Safety-Enhancing Strategy

**DOI:** 10.3390/s24237672

**Published:** 2024-11-30

**Authors:** Xiao Liang, Chuan-Zhi Thomas Xie, Hui-Fang Song, Yong-Jie Guo, Jian-Xin Peng

**Affiliations:** 1 School of Civil Engineering and Architecture, Wuhan Institute of Technology, Wuhan 430205, China; 13333494619@163.com (X.L.); 15623873962@163.com (Y.-J.G.); 2 Yangtze Delta Region Academy of Beijing Institute of Technology, Jiaxing 314019, China; xiechuanzhi@buaa.edu.cn

**Keywords:** intelligent transportation systems (ITSs), onboard vehicle sensors, Three-Stage Cellular Automata (TSCA) model, roundabout traffic optimization

## Abstract

Intelligent transportation systems (ITSs) present new opportunities for enhanced traffic management by leveraging advanced driving behavior sensors and real-time information exchange via vehicle-based and cloud–vehicle communication technologies. Specifically, onboard sensors can effectively detect whether human-driven vehicles are adhering to traffic management directives. However, the formulation and validation of effective strategies for vehicle implementation rely on accurate driving behavior models and reliable model-based testing; in this paper, we focus on large roundabouts as the research scenario. To address this, we proposed the Three-Stage Cellular Automata (TSCA) model based on empirical observations, dividing the vehicle journey over roundabouts into three stages: entrance, following, and exit. Furthermore, four optimization strategies were developed based on empirical observations and simulation results, using the traffic efficiency, delay time, and dangerous interaction frequency as key evaluation indicators. Numerical tests reveal that dangerous interactions and delays primarily occurred when the roundabout Road Occupancy Rate (ρ) ranged from 0.12 to 0.24, during which times the vehicle speed also decreased rapidly. Among the strategies, the Path Selection Based on Road Occupancy Rate Recognition Strategy (Simulation 4) demonstrated the best overall performance, increasing the traffic efficiency by 15.65% while reducing the delay time, dangerous interactions, and frequency by 6.50%, 28.32%, and 38.03%, respectively. Additionally, the Entrance Facility Optimization Strategy (Simulation 1) reduced the delay time by 6.90%. While space-based optimization strategies had a more moderate overall impact, they significantly improved the local traffic efficiency at the roundabout by approximately 25.04%. Our findings hold significant practical value, particularly with the support of onboard sensors, which can effectively detect non-compliance and provide real-time warnings to guide drivers in adhering to the prescribed traffic management strategies.

## 1. Introduction

In the current era of rapid urbanization and technological advancements, intelligent transportation systems (ITSs) are crucial for managing the growing complexity of modern traffic networks. With the rising number of vehicles, governments worldwide are integrating advanced technologies, including artificial intelligence (AI), the Internet of Things (IoT), and vehicle-to-everything (V2X) communication. Among these, onboard sensors play a pivotal role, seamlessly merging with urban infrastructure to form a comprehensive data network that captures detailed, real-time information. These sensors, integrated within ITS frameworks, provide critical inputs on vehicle speed, position, and environmental conditions, contributing to dynamic traffic predictions and enabling adaptive traffic control measures. Through such in-vehicle and infrastructure-based sensor networks, ITS enhances traffic management, boosts safety, and reduces congestion by facilitating data-driven decision-making processes [1]. In China, policies under the “New Infrastructure” initiative are being emphasized as essential for the development of smart cities and intelligent transport solutions, with investment in smart transportation and related IoT growing by 34.1% in the first half of 2023 [2]. These technologies are vital for tackling the challenges of congested roads and dynamic traffic conditions. Intelligent transportation systems necessitate a profound understanding of the complex interactions between driver behavior, vehicle dynamics, and infrastructural design. This is particularly evident in the case of roundabouts, where vehicle movement and decision-making are intricately shaped by factors such as vehicle positioning, congestion levels, and the geometric configuration of the roundabout [3]. Understanding vehicle motions at roundabouts, along with how these characteristics impact drivers’ decisions (e.g., gap acceptance, yielding, and exit choices), is critical for optimizing traffic flow and enhancing safety through ITS-based solutions.

Currently, researchers mostly resort to two approaches to this end: experiments, in which real system behavior characteristics can be extracted; and simulations, which effectively replicate and collect data, and explore scenarios that are hard to investigate through experimental research. Recent studies demonstrate that simulations play a crucial role in guiding practical projects, validating model outcomes, and ensuring effective implementation in engineering applications [4,5,6,7,8,9]. This interplay between simulation and real-world execution underscores the importance of integrating theoretical insights with practical experience. For example, researchers have conducted empirical studies of vehicle behavior (e.g., gap-acceptance behavior, aggressive driving behavior, etc.) in roundabout traffic flow, aiming to establish a reliable experimental basis for proposing, validating, and evaluating corresponding traffic flow models [10,11,12]. The variations in vehicle behavior depending on different positions in a roundabout (e.g., entrance, exit, etc.), as well as across different types of roundabouts (e.g., multi-lane roundabouts, turbo roundabouts, etc.), have also been explored [13,14,15,16]. All these efforts certainly deepen our understanding of the actual vehicle motion characteristics in roundabouts and may lead to the development of better traffic flow guidance measures for transportation spaces.

However, in reality, traditional roundabouts continue to face significant challenges, especially in larger ones, where congestion and accidents remain frequent, and existing solutions have proven inadequate. A study comparing two-lane roundabouts to traditional signalized intersections found that congestion was more severe in roundabouts when subjected to high traffic volumes (approximately 2.44 vehicles per second) [17]. The three-lane Lihejiang Roundabout in Qinzhou City, Guangxi Province, frequently experiences traffic jams lasting over half an hour during peak periods in the morning and evening [18]. Considering these issues, what factors contribute to the higher level of confusion at large roundabouts that current modeling tools struggle to adequately replicate. At least three reasons contribute to this situation: (a) large roundabouts provide vehicles with greater maneuvering space, leading to more diverse vehicle behaviors and an increased interaction frequency between vehicles; (b) vehicles entering the roundabout from different entries exhibit significant heterogeneity in route choice attributes; (c) compared to intersections, large roundabouts typically have a more complex spatial layout, which places higher demands on drivers’ decision-making. Drivers need to accurately time their exit from the roundabout; otherwise, they may miss their exit or cause congestion. Given these persistent challenges, this study aimed to investigate the underlying causes of high congestion and accident rates at large roundabouts, with a focus on understanding the factors that contribute to complex vehicle interactions and driver decision-making processes in these environments. This research addressed the following questions: (a) What specific traffic dynamics contribute to elevated congestion levels at large roundabouts? (b) How do the unique spatial characteristics of large roundabouts influence driver behavior and decision-making? (c) How can roundabout traffic flow efficiency be effectively optimized?

By employing observational and statistical analysis methods, Cheng et al. [19] used Changchun’s large roundabout as a case study to examine vehicle behavior. Similarly, Xie et al. [20] adopted a comparable approach, developing a fine-grid cellular automaton (CA) model to describe individual behaviors and explore the effects of specific behaviors on individual movement in a space. Ruili Wang et al. [21] focused on vehicle behavior in the entry and exit areas of multi-lane roundabouts and conducted a study on the throughput in these regions. And Jing Bie et al. [22] paid attention to drivers’ lane choice patterns, for the analysis of the capacity of a multi-lane roundabout. Tumminello et al. [23] conducted a study integrating roundabouts into smart mobility frameworks, focusing on enhancing safety and efficiency in connected and autonomous vehicle (CAV) environments. Through traffic simulations, they examined how vehicle-to-everything communication and sensors contribute to smoother traffic flow and reduced collisions. Similarly, Gan et al. [24] explored decision-making models for autonomous vehicles at unsignalized roundabouts, using deep reinforcement learning algorithms. Their research emphasizes the importance of sensors in providing spatial perception data for path planning and safety enhancement. Although the above studies have provided valuable insights into large roundabouts, when considering vehicle decision-making and interactions at large roundabouts, a more detailed traffic flow model is required, i.e., each vehicle be described more precisely. This paper presents several innovations that address this research gap regarding traffic flow efficiency and complex traffic dynamics in large roundabout systems:

- A roundabout traffic flow model is proposed to replicate the complex interactions between vehicles in large roundabouts, simulating three stages: entrance, following, and exit.
- A multi-lane vehicle motion model for large roundabouts was developed.
- A study was conducted on the traffic flow efficiency in large roundabouts.
- Multiple optimization strategies for enhancing traffic efficiency are proposed, based on the spatial and vehicular behavior characteristics of large roundabouts.

The objective of this study was to develop a model that accurately replicates the complex traffic flow dynamics in large roundabouts. It aimed to assess the influence of driving behaviors on traffic flow efficiency, focusing on how these behaviors affect overall congestion levels and the smoothness of traffic. Furthermore, this study sought to propose and evaluate optimization strategies to enhance traffic efficiency.

The rest of this paper is organized as follows: We begin with Section 2, where we report our careful field observation of the Guanggu Roundabout and detailed empirical analysis. These observations provided data support for establishing a micro-traffic flow model for the roundabout, which led to the development of a three-stage cellular automata (TSCA) model. Section 3 is dedicated to the quantitative comparison between model predictions and empirical results. Finally, in Section 4, we report how four optimization strategies and guiding measures were applied within the model and tested numerically.

## 2. Model

In this section of the study, we collected observational statistics of the roundabout traffic flow and identified empirical findings. Based on these findings, we constructed a simulation model using the Cellular Automata approach.

### 2.1. Empirical Research

#### 2.1.1. Research Scenario

To analyze the operational characteristics of traffic flow over a complex roundabout environment, we conducted a field study at the Guanggu Roundabout in Wuhan, China, for the following reasons: (i) this roundabout ranks as the largest in Asia in terms of dimensions (e.g., number of lanes, entries, and exits) [25], leading to more intricate and diverse vehicle interactions and driver behaviors when compared with smaller ones; and (ii) the maximum peak-hour traffic volume reaches 10,000 vehicles [26], offering a broad spectrum of density conditions, which is essential for comprehensive research.

**Figure 1:**
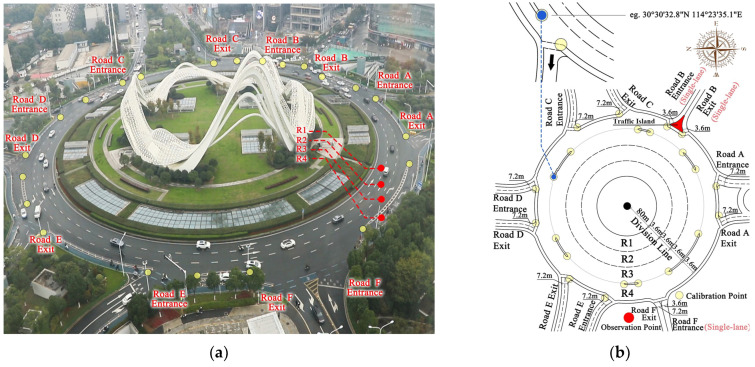
Snapshot and schematic view of the Guanggu Roundabout, where (**a**) is a snapshot from the observation point, taken on 11 November 2023, and (**b**) is the schematic sketch providing detailed spatial information based on (**a**).

To find an unobstructed and elevated filming vantage point, we carefully selected observation points for our field study (Figure 1a gives a recording snapshot). Spatially, the Guanggu Roundabout consists of four concentric lanes (denoted R1 to R4 from inner to outer), which are interconnected with the external traffic network through several radial roads. For clarity in our descriptions, we named these radial roads Road A to Road F in the counterclockwise direction (note that each road has both exit and entrance branches). With the assistance of Google Maps, the geometry of the roundabout was sketched, as shown in Figure 1b, where one may notice, i.e., (i) the lane widths of R1 to R4 are uniformly 3.6 m, with circumferences of 514 m, 536 m, 559 m, and 581 m, respectively; (ii) the Road F entrance and Road B entrance and exit each contain one lane, whereas the entrances and exits of the other radial roads contain two lanes (although the exit of Road F contains two lanes, pedestrians often occupy one lane owing to the lack of a sidewalk, thereby limiting the exit to accommodating only one vehicle simultaneously); (iii) precise latitude and longitude information of the calibration points (see the zoomed-in inset in Figure 1b), typically with distinct features (e.g., the start and end points of channelizing islands and the intersection points of R4 with Roads A–F), has been selected to accurately depict the locations of entrances and exits relative to the roundabout and determine vehicle positions for further analysis (reported in Section 4). Given the research goal of exploring the dynamics of traffic flow over the roundabout, we only considered the junctions of the radial roads with the roundabout (i.e., as the input sources of vehicles) rather than considering the entire segment of the radial roads.

#### 2.1.2. Empirical Analysis

To record traffic flow data at the roundabout, a camera (camera details: brand: Canon 6D Mark II; manufacturer: Canon Inc., Tokyo, Japan; resolution: 1080P; fps: 25) was installed at the observation point (i.e., the place captured in Figure 1a), positioned approximately 60 m high, enabling comprehensive recording of the traffic flow over the roundabout. The quantitative data, including the vehicles’ entrance and exit times, travel distances, path selection, and the total number of vehicles on the roundabout at various observation times, were manually extracted from the videos through visual inspection.

The observation period spanned from 2:12:32 p.m. to 2:32:32 p.m. on 11 November 2023, totaling 1200 s. During this time, 1507 vehicles traversed the roundabout, exhibiting certain common driving behaviors, which were the focus of our empirical analysis. To gain deeper insights, we tracked the routes and decisions of vehicles, identifying three movement stages: entrance, following, and exit stages. Additionally, we analyzed the entrance and exit radial roads (i.e., Roads A–F in Figure 1) for each vehicle and defined ΔR as the entry–exit distance (e.g., if a vehicle enters at Road A and exits at Road C, then ΔR=2) to explore the impact of relative entry–exit locations on lane choice decisions. On the basis of these observations, we can make the following empirical assertions, categorized by stages.

When vehicles occupy the junction between the radial road and the roundabout, the entrance stages for these vehicles begin:

(1) There is a distinct relationship between ΔR and lane choice behavior, as described in the Table 1 and Table 2. Specifically, vehicles with smaller ΔR are more likely to choose lanes farther from the center of the roundabout (e.g., when ΔR=1, vehicles tend to choose R3 or R4); conversely, as ΔR increases, vehicles are more likely to select lanes closer to the center (e.g., when ΔR>2, vehicles tend to choose R1).
(2) Vehicles engage in continuous lane changing from their initial entry positions, and the closer the target lane is to the center of the roundabout, the longer the required driving distance to complete this process (i.e., before starting the following stage). Qualitatively, the average driving distances for vehicles targeting lanes R1, R2, and R3 are approximately 28 m, 16 m, and 10 m, respectively (the straight-line distance between the initial entry position and the final position, where the vehicle is fully aligned in the target lane, is defined as the driving distance; these positions are measured using calibration points (Figure 1) and supplementary location data from Google Maps, including distinctive markers (e.g., lane markings, streetlights) surrounding the vehicle); vehicles with R4 as the target lane do not exhibit lane-changing behavior.
(3) Before initiating continuous lane changing, vehicles must check for the traffic status behind the perpendicular line formed by the initial entry positions and the target lane. The lane-changing process can only begin if other vehicles behind this line are at distances approximately greater than 24 m, 17 m, and 13 m for R1, R2, and R3, respectively.
(4) During the lane-changing process, successful execution can only occur if the distance between other behind vehicles and the intended position of the lane-changing vehicle in the target lane exceeds 12 m.
(5) For the entrances of Roads A, C, D, and E, which contain two lanes, when two vehicles are simultaneously waiting to enter the roundabout, the vehicle on the right moves first if both are competing for the same position.

When vehicles complete the entrance stage, the following stages begin:

(6) Vehicles tend to maintain their current lane throughout the following stage unless they encounter congestion. In the event of congestion, vehicles in R3 and R4 typically remain in their current lane, while approximately 60% and 45% of vehicles in R1 and R2, respectively, will choose to change lanes.
(7) Similar to (4), for vehicles in the following stage intending to change lanes, the lane change can only be executed if the distance between the vehicles behind in the target lane and the intended position of the lane-changing vehicle is greater than 12 m.
(8) Except for lane changes due to congestion, about 86% of vehicles in R1 will change to R2 in advance when there is one exit remaining between their current position and the target exit, in preparation for leaving the roundabout.

For vehicles positioned such that their nearest exit road in the driving direction is the intended exit, the exit stages commence:

(9) For vehicles in R1 and R2 intending to exit, difficulties may arise in executing continuous lane-changing behaviors due to the surrounding traffic flow, which primarily consists of vehicles in the following stage moving at relatively high speeds. Instead, they typically follow a pattern of lane change→drive straight→lane change, to move from inner lanes (i.e., R1 and R2) to outer lanes (i.e., R3 and R4). Specifically, the conditions for lane changing must adhere to Assertion (7), and the follow-up drive-straight distance after a lane change is approximately 4 m.
(10) For vehicles in R3 and R4, they have direct access to exits, and if their target exit roads are with two lanes (i.e., Roads A, C, D, and E), they tend to choose the exit lane closer to their current position.
(11) If conflicts arise when vehicles in R3 and R4 compete for the same position on the exit road, the vehicle in R4 typically secures the intended position successfully. In contrast, the vehicle in R3 will either opt for the other, more distant exit lane on the same road or wait until the intended closer exit lane becomes available.
(12) Vehicles in R1, R2, and R3 typically travel 32 m, 18 m, and 10 m, respectively, to exit the roundabout (note that the distance is measured from the initial position at the start of the exit process to the final position on the exit road, similar to the definition in point (2)). Vehicles in lane R4, however, continue along R4 until the intended exit position becomes available.

Albeit interesting per se, these observations hardly suffice to gauge the safety and efficiency of the collective vehicle flow or explore the effect of variations in spatial settings or driving behavior guidance strategies. Therefore, we turn to the development of a model premised on the empirical findings, but with broader applicability.

### 2.2. Introduction of a Three-Stage Cellular Automata (TSCA) Model

Cellular Automata (CA) models, known for their scalability and computational efficiency, are extensively utilized in traffic flow dynamics research to investigate traffic flow efficiency, offering insights into flow patterns and dynamics. Among these, the NaSch model [27,28,29,30,31] is particularly recognized for its effectiveness in describing car-following behaviors, making it ideal for simulating complex interactions and optimizing flow efficiency across various traffic scenarios. Marzoug et al. [32] examined traffic flow efficiency at conventional traffic lights, double traffic lights, and self-organizing intersections. Wang et al. [33] used Cellular Automata models and a mixed NaSch traffic flow model to analyze traffic flow characteristics, finding that the maximum speed of short vehicles significantly impacts overall traffic flow efficiency when the mixing ratio and vehicle length are fixed. Qiao et al. [28] studied traffic density and congestion patterns in two typical Cellular Automata traffic models (VDR and TT models) with slow-to-start rules, under both periodic and open boundary conditions. For periodic boundary systems, the initial conditions significantly influenced congestion formation and flow efficiency, while for open boundary systems, the exit probability played a major role in traffic congestion and flow stability. Lakouari et al. [34] proposed a Cellular Automata model to describe driver behavior at a single-lane urban roundabout and explored the differences in flow efficiency between single-lane roundabouts and signalized intersections. Cellular Automata models offer a robust and adaptable framework for simulating complex traffic interactions, making them ideal for evaluating and optimizing traffic flow efficiency in various settings. Given the intricate spatial characteristics of large roundabouts and the multifaceted nature of vehicle movement behaviors across three stages (i.e., entering, following, and exiting), the NaSch model alone is insufficient for comprehensive modeling. To address this, we propose a Three-Stage Cellular Automata (TSCA) model, which extends the NaSch model to accurately depict car-following behaviors while incorporating additional rules tailored to the distinct vehicle behaviors observed at each stage, thereby enhancing the simulation accuracy of dynamic interactions within the roundabout environment.

Overall, in the context of vehicle movement, the basic assumptions of our TSCA are derived mostly from the following general principles, along with the empirical observations:

(1) Vehicles cannot overlap in space.
(2) Each vehicle is assigned a different desired speed and occupies two cells in its direction of movement (note: the definition of cells is detailed in Section 2.2.1).
(3) Each vehicle needs to undergo entrance, following, and exiting stages, with movement rules and features varying across these stages (Section 2.2.4, Section 2.2.5 and Section 2.2.6).
(4) Vehicle lane-changing behavior is detailed as encompassing both vertical and horizontal movements relative to the direction of travel; thus, this behavior impacts a broader area of the neighborhood compared to simple following behavior.
(5) For each position update, a vehicle’s maximum movement distance is limited to one cell.
(6) When vehicles compete for the same intended position simultaneously, those in the following stage take priority over those in entrance and exit stages. If more than two vehicles in the same movement stage are competing, the order of their updates is determined randomly.

While the above assumptions provide a fundamental basis for vehicle movement, they are not sufficiently refined for microscopic modeling. Therefore, a more detailed description of our TSCA is presented below to capture the intricacies of vehicular behavior at the roundabout.

#### 2.2.1. Transformation of the Guanggu Roundabout into a Modeling Scenario

**Figure 2:**
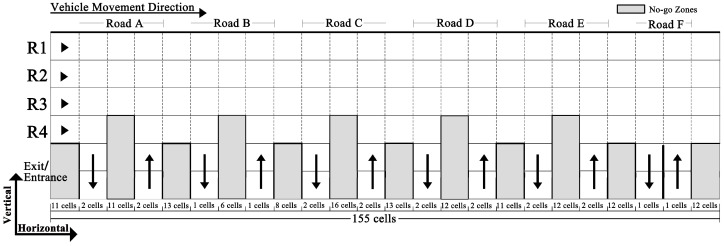
Illustration of discretizing the Guanggu Roundabout into a scenario made of cells for TSCA modeling.

Similar to Huang [35], for Cellular Automata modeling, we transformed the circular structure of the Guanggu Roundabout into a rectangular modeling scenario (the Division Line serves as the starting point for unfolding the roundabout into a rectangular form, in Figure 1b), which includes four straight lanes, each consisting of multiple rectangular cells, corresponding to the four circular lanes (R1 to R4) in reality; the entrances and exits of the radial roads (Roads A to F) are also represented as one or two rectangular cells, each capable of accommodating one or two vehicles (Figure 2). If comparing Figure 1 and Figure 2, one can observe that lanes closer to the center of the roundabout have shorter lengths (in the vehicle movement direction), prompting us to adjust the lengths of cells according to their location on R1 to R4. Specifically, we adopt 3.6 m, widely used in the literature (e.g., Li et al. [13]), as the reference length for each cell. Given the total lengths of lanes R1 to R4, the detailed configurations are as follows: (i) each lane is discretized into 1 × 155 cells; (ii) cell lengths along the vehicle movement direction for R1 to R4 are set at 3.32 m, 3.46 m, 3.6 m, and 3.75 m, respectively, ensuring the lanes’ total lengths are approximately 514 m, 536 m, 559 m, and 581 m, respectively; (iii) the cell widths for R1 to R4 are standardized at 3.6 m, reflecting their actual dimensions. In addition, with a standardized cell size of 3.6 × 3.6 m, the entrances and exits of the radial roads are abstracted as either 2 × 2 cells or 2 × 1 cell structures, depending on the number of lanes present in Roads A to F.

Transforming the roundabout into a rectangular-shaped scenario inevitably introduces edge effects. To allow vehicles entering from Roads D, E, and F to exit via Roads A, B, and C in Figure 2, we artificially connect the right edge of Figure 2 to the left edge, by treating cells around the right and left edges as neighbors, ensuring continuous vehicle flow across the edges.

#### 2.2.2. A Sequential Update Scheme with Stage-Aware Priority Orders

For discrete modeling multi-agent complex systems (e.g., vehicles [36] and pedestrians [5,20,27,37]), sequential update schemes are commonly employed. When such schemes are utilized, two critical pre-settings must be established: the update time interval (Δt), and the sequence order for agent updates. With respect to vehicle flow, for one thing, Laarej et al. [38] adeptly characterized vehicles with two speed types (fast and slow) by adhering to the common setting of Δt=1 s in their NaSch CA model [39]; additionally, Regragui and Moussa [35] established location-based rules for assigning vehicle priority in predefine conflict zones to define the sequence order.

Drawing inspiration from Laarej et al. [38] and Regragui and Moussa [40], and incorporating our field study findings, we further defined vehicles with a wider range of velocities by adopting a smaller Δt=0.01 s (i.e., the interval between time steps (Ts) is set as 0.01 s) for system time iterations; meanwhile, the sequence order for vehicle updates at the same Ts is determined in the basis of their current movement stages. Specifically, at each Ts, vehicles needing updates are categorized into two waiting sequences: those in the following stage (ws-1) and those in the entrance/exit stage (ws-2). Vehicles in ws-1 have priority and are updated first in a randomly shuffled order. Once ws-1 updates are complete, ws-2 vehicles are updated similarly. After updating, intervals before the next update are calculated on the basis of vehicle velocities (as detailed in Section 2.2.3). This sequential update process continues until all vehicles exit the roundabout.

#### 2.2.3. Basic Model of Vehicle Movement

When vehicles move around the roundabout, most are engaged in the car-following stage. Therefore, we utilize the NaSch model [30] as the foundation for our TSCA. Nonetheless, the standard NaSch model, with its uniform update interval, falls short of capturing the diverse range of vehicle velocities observed in our empirical study. To address this, TSCA introduces dynamic update time intervals based on individual vehicle velocities, allowing for a more precise and nuanced representation of varying velocity statuses.

Assuming vehicle i executes a movement at time t (unit: s) with a speed of $v_{i}(t)$ (measured in cell/s), the interval $\delta_{t}^{i}$ (unit: s) between the current movement and its next movement is calculated as follows:

(1) $$\delta_{t}^{i}=\left\{\begin{array}{cc} \frac{k}{v_{i}(t)} & \mathrm{if}\;v_{i}(t)>0\\ 1 & \mathrm{if}\;v_{i}(t)=0 \end{array}\right.$$

where *k* reflects the cell’s length/width. Numerically, for vehicles moving horizontally (Figure 2) in R1, R2, R3, and R4, the values of *k* are set as 0.92, 0.96, 1, and 1.04, respectively, aligning with the cell lengths in R1 (3.32 m), R2 (3.46 m), R3 (3.6 m), and R4 (3.75 m) relative to the reference length of 3.6 m; for vertical movements, the width of each cell is 3.6 and thus *k* is set as 3.6/3.6 = 1. t is computed as Ts×Δt according to the TSCA’s time discretization scale. The value of $\delta_{t}^{i}$, as determined by Equation (1), is rounded to two decimal places, and thus t can only increase by 0.01 (i.e., in line with Δt) instead of smaller time intervals during implementation.

After determining $\delta_{t}^{i}$, vehicle i needs to update its speed at $t+\delta_{t}^{i}$ during t→$t+\delta_{t}^{i}$, based on the following rules extended from the NaSch model.

Rule 1: acceleration, i.e., $v_{i}(t+\delta_{t}^{i})=\min(v_{i}(t)+1,v_{\max}^{i})$.

Rule 2: deceleration, i.e., $v_{i}(t+\delta_{t}^{i})=\min(v_{i}(t+\delta_{t}^{i}),G_{f}^{c}(i)/\delta_{t}^{i})$.

Rule 3: randomization, i.e., $v_{i}(t+\delta_{t}^{i})=\max(v_{i}(t+\delta_{t}^{i})-1,0)$, with a braking probability *p*.

**Figure 3:**
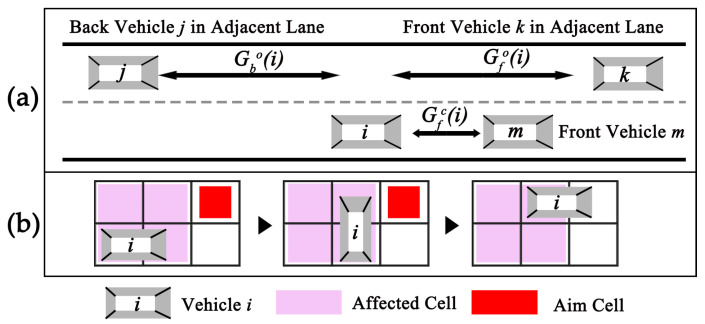
(**a**) Illustration of variables related to determining the updating position of vehicle iii, with $G_{f}^{o}(i)$, $G_{b}^{o}(i)$, and $G_{f}^{c}(i)$ denoting the number of cells horizontally from the nearest vehicles surrounding vehicle i; (**b**) demonstration of the lane-changing process of vehicle i and the affected area during such behavior.

In Rules 1–3, $v_{\max}^{i}$ and $G_{f}^{c}(i)$ denote the maximum speed and the number of empty cells in front of vehicle i, respectively (Figure 3a).

If vehicle i is in the car-following stage without lane-changing behaviors, it typically updates its position one cell forward horizontally at time $t+\delta_{t}^{i}$. Given that there are vehicles needing to change lanes, although the time interval and velocity for such behavior are also calculated using Equation (1) and Rules 1–3, the movement rules require further definition: (i) vehicles perform vertical movement first, then move horizontally; (ii) lane-changing behavior affects four cells, and other vehicles cannot occupy these cells during this process (Figure 3b).

This section provides general update time intervals and movement rules, based on which vehicles enter the roundabout from various road entry points in the system and proceed through a sequence of updates across three consecutive stages before exiting. During this process, they must adhere to distinct movement rules specific to each stage—entrance, following, and exit. Further elaboration on these specific movement dynamics in each stage is provided in Section 2.2.4, Section 2.2.5 and Section 2.2.6 to complete the construction of TSCA.

#### 2.2.4. Motion on the Entrance Stage

**Figure 4:**
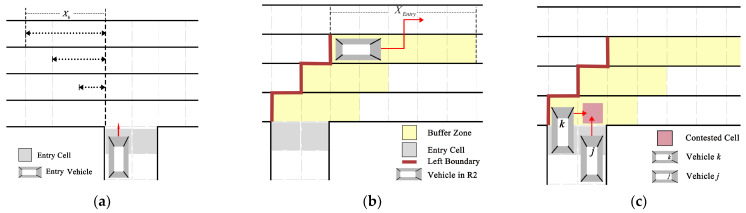
Illustration of (**a**) a vehicle waiting in the entry cell; (**b**) the trajectory of the vehicle as it moves from R2 to R1; (**c**) two vehicles competing for the same cell.

Once the vehicle occupies the entry cell, it transitions to the entrance stage. At this stage, vehicles occupy the entry cell and then continuously change lanes until they reach the target lane, with each vehicle being updated at the respective update time step. Based on the basic movement model, the entrance stage rules are as follows:

(1) Entry vehicles in the entry cell need to ensure, before initiating continuous lane changes, that there are no other vehicles within the distance $X_{b}$ behind the perpendicular line formed by the entry vehicle and the lanes between the target lane (Figure 4a). In R1, R2, and R3, this distance $X_{b}$ is four, six, and seven cells, respectively. R4 is not considered in this scenario.
(2) Vehicles coming from the entry cell enter R4 and then move within the buffer zone (shown as the yellow area in Figure 4b) in the entrance stage. For a dual-lane entrance (i.e., entrance of Road A, Road C, Road D, Road E), the cell number $X_{Entry}$ of the buffer zone in different lanes is as follows: seven cells (R2), four cells (R3), and three cells (R4). For a single-lane entrance (i.e., entrance of Road B, Road F), the value is decreased by one. The position of the buffer zone is determined by the left boundary (Figure 4b).

In the entrance stage, the following rules extend the basic movement model:

(3) In the buffer zone, each time vehicles complete a lane-changing behavior, if they have not yet entered the target lane, they prioritize continuing to change lanes rather than moving horizontally at the next update time step. However, when a vehicle completes a lane change to enter R2 and the target lane is R1, it needs to move one cell horizontally before choosing a lane-changing behavior again (Figure 4b).
(4) For the same aim cell at the same update time step, for the dual-lane entry (i.e., entrance of Road A, Road C, Road D, Road E), the vehicle in the right entry cell has priority to occupy that aim cell (shown as the vehicle j in Figure 4c). Other vehicles, preparing to move horizontally and complete a lane-changing behavior, will wait until the competition is resolved (shown as the vehicle k in Figure 4c).
(5) In the case of a vehicle preparing to change lanes in a vertical movement at the update time step, it needs to consider the position and movement status of other vehicles in the adjacent lane behind it. Specifically, when the distance $G_{b}^{o}(i)$ is greater than three cells (Figure 3a) and the adjacent cell in the other lane is unoccupied, the vehicle can change lanes vertically at this update time step.

#### 2.2.5. Motion in the Following Stage

**Figure 5:**
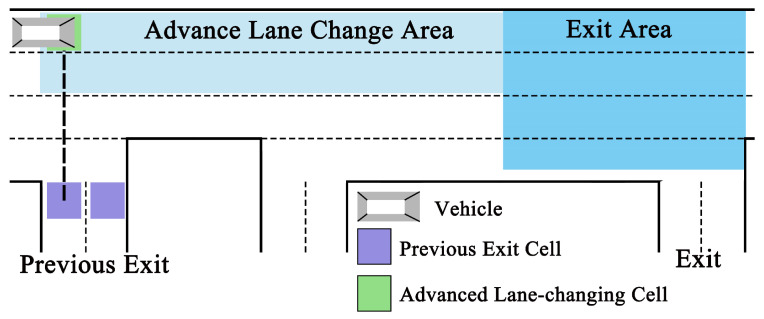
Demonstration of a scenario where a vehicle is preparing to exit the roundabout during the following stage.

Upon entering the target lane and transitioning to the following stage, the vehicle primarily moves horizontally, changing lanes only in response to congestion or at designated locations until it reaches the exit area (Figure 5). The vehicle updates its position at each respective time step according to the sequential update algorithm. Although the vehicle’s movement is governed by the basic movement model, additional rules are introduced in this stage to refine its behavior, ensuring accuracy in the simulation of the traffic flow:

(1) For the vehicle, if it has remained stationary for two consecutive updates, there is a 60% chance of it changing lanes if it is in R1, a 45% chance if it is in R2 (with a 5% chance of moving to R1, and a 40% chance of moving to R3), and the vehicle will remain in its current lane if it is in R3 or R4. Lane changing will not be chosen if the adjacent cell in the other lane is occupied.

(2) When the vehicle in R1 passes the advanced lane-changing cell (shown as the green block in Figure 5), there is an 86% chance that it will opt to switch to R2 in subsequent movements; otherwise, it will proceed straight. For the single-lane previous exit (i.e., exit of Road B, Road F), advanced lane-changing cells are positioned in R1 at locations aligned with the previous exit cell; for a dual-lane previous exit (i.e., exit of Road A, Road C, Road D, Road E), they are aligned with the left exit cell (shown as the purple block in Figure 5).
(3) In the following stage, the vehicle cannot process a vertical movement of lane change at the update time step unless the distances $G_{b}^{o}(i)$ and $G_{f}^{o}(i)$ are greater than three and two cells, respectively (Figure 3a).

After the vehicle reaches the area of the exit stage, it exits the following stage.

#### 2.2.6. Motion in the Exit Stage

**Figure 6:**
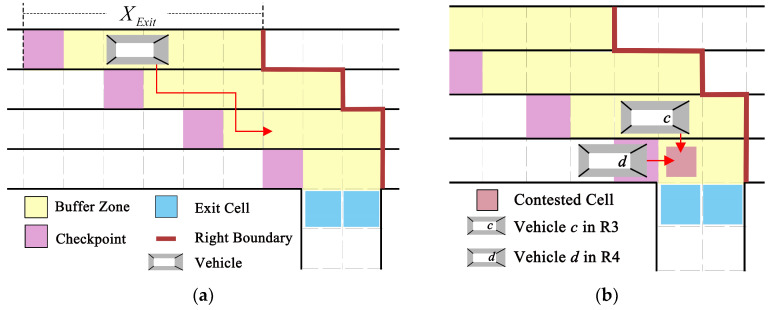
(**a**) Lane-changing rules for vehicles at the exit stage; (**b**) illustration of two vehicles competing for the same cell during the exit stage.

For vehicles reaching the checkpoint (shown as the pink block in Figure 6a), they transition to the exit stage, initiating a lane-changing movement towards the exit cell. Vehicles moving during the exit stage require additional modeling rules, as detailed below:

(1) Vehicles coming from the following stage enter the checkpoint of the exit stage, and then they move within the buffer zone (shown as the yellow area in Figure 6a). $X_{Exit}$ is the cell number of the buffer zone in different lanes. For dual-lane exit (i.e., exit of Road A, Road C, Road D, Road E), the values are as follows: 10 cells (R1), 7 cells (R2), 5 cells (R3), and 3 cells (R4); for single-lane exit (i.e., exit of Road B, Road F), the values are decreased by one. The position of the buffer zone is determined by the right boundary (Figure 6a). The checkpoint is the left boundary cell of the buffer zone, and the position is determined by the length of $X_{Exit}$ in different lanes.
(2) When vehicles change lanes towards the exit from R1 and R2, they must move horizontally at least two cells after completing a lane-changing behavior before they can change lanes again (shown as the vehicle in Figure 6a). At this stage, the conditions required for the vehicle to change lanes are the same as those in the following stage.
(3) For the same aim cell at the same time step, the vehicle in R4 to the exit cell has priority to occupy that cell over other vehicles (shown as vehicle d in Figure 6b). Other vehicles, in R3, will wait until the competition is resolved (in single-lane exit) or detour (in dual-lane exit) to exit the system (shown as vehicle c in Figure 6b).

After the vehicle enters the exit cell, it is removed from the system.

## 3. Validation of the Model and Results

We tested and validated the TSCA model by comparing its results to the data extracted from our empirical observations, in MATLAB R2023b. The total simulation duration was 20 min, with an injection rate of 0.75 s for each entrance. Additionally, for vehicles, we converted the observed data into probabilities to support the selection of the aim exit and the target lane (refer to Appendix A).

In this section, two indicators are adopted to compare the model output with that of the empirical study: (1) the fundamental diagram, which is one of the most characteristic features in roundabout traffic flow studies, and (2) the evolution of the outflow number. Additionally, we conducted further research on the phenomena and issues that appeared during the simulation.

### 3.1. Fundamental Diagram

**Figure 7:**
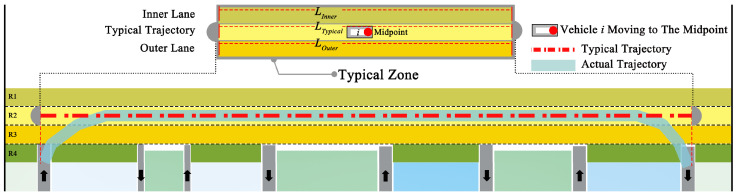
Variables used in the typical trajectory.

We plotted the ‘fundamental diagram’ associated with the vehicles (in the simulation) on the basis of their movement over the roundabout. Owing to the difficulty in determining the actual vehicle paths, we established a typical trajectory to standardize the vehicle movement distance between the simulation and real-world conditions (Figure 7). The typical trajectory is the portion of the vehicle’s movement trajectory projected perpendicularly onto the target lane (as shown by the red and blue lines in Figure 7). In real-world scenarios, similarly, the endpoints of typical trajectories are marked via Google Maps, with the trajectory length measured as the arc length between these points in meters. The total duration $T_{d}$ of the vehicles on the roundabout is calculated by the video timestamps of entering and exiting the roundabout in seconds (in the simulation, this is recorded by the system).

In Figure 7, $L_{Innert}$, $L_{typical}$, and $L_{Outer}$ denote the lengths (m) of the inner lane, typical trajectory, and outer lane, respectively. These are obtained by multiplying the number of cells by the length represented by each cell.

We introduce a new parameter, the Road Occupancy Rate (ρ), which represents the proportion of road surface area occupied by vehicles in relation to the total available road surface area. The fundamental relationship is obtained by averaging vehicles’ ‘effective velocity’ under the same ρ, to facilitate the calculation and provide a more accurate representation of utilization and traffic dynamics. For each vehicle, the effective velocity $V_{e}$ (m/s) is defined by the length of the typical trajectory $L_{typical}$ and the total duration $T_{d}$ of each vehicle within the system, that is,

(2) $$V_{e}=\frac{L_{typical}}{T_{d}}$$

For the calculation of ρ, we performed an analysis based on the typical zone (consists of the inner lane, typical trajectory, and outer lane in Figure 7). ρ is defined as the ratio of the total vehicle area $S_{Agent}$ the total area of the zone $S_{Zone}$, as follows:

(3) $$\rho =\frac{S_{Agent}}{S_{Zone}}$$

Here, for each vehicle, the total vehicle area $S_{Agent}$ represents the area occupied by all vehicles within the typical zone, when the front of the vehicle reaches the midpoint of the typical trajectory. Due to the complex dimensions of vehicles on the roundabout and the fact that the road can accommodate only one vehicle at a time, we assumed each vehicle to be a rectangle with a length of 5 m and a width of 3.6 m.

**Figure 8:**
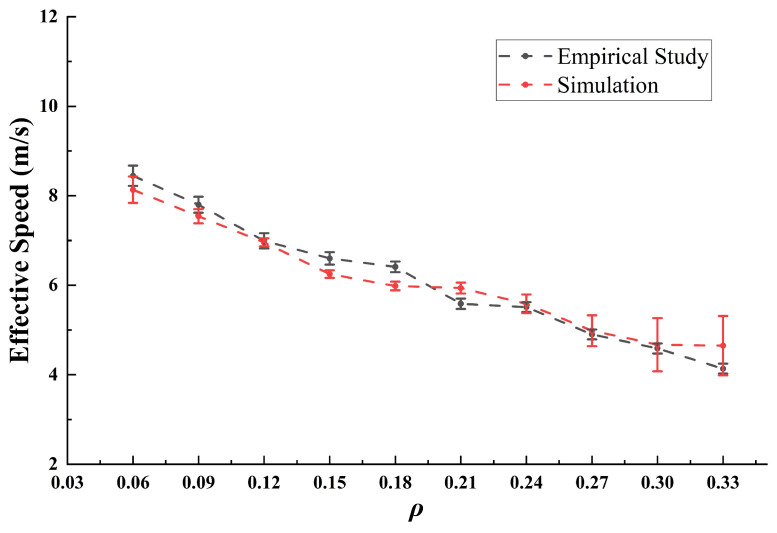
Fundamental relationship between the empirical and simulation results. For ρ, the range is determined to be 0.03 to 0.33, which is statistically significant. In other ranges, the sample size is too small to effectively reflect the characteristics of traffic flow.

On that basis, we plotted the fundamental diagram (Figure 8). It can be noted that the data values extracted from empirical research (gray line) closely approximate those in the simulation (red line). As ρ increases, the effective speed decreases in a similar manner. With a high ρ, the consistency between the two curves is more satisfactory. However, at a lower ρ, slight differences can be observed between the simulation data and empirical research, although overall consistency remains strong.

To better validate the performance of the fundamental diagram, linear regression was employed to test the data. We compared the empirical and simulated effective speeds under the same ρ, using the empirical speed as the independent variable (i.e., *y* in Equation (4)) and the simulated one as the dependent variable (i.e., *x* in Equation (4)) based on the processed data. The goodness of the linear fit, evaluated with $R^{2}=0.9706$, and the resulting equation were formulated as follows:

(4) y=0.8325x+0.99341

Here, the data are effective and exhibit a clear trend. The fundamental diagram reflects the global dependency of traffic on ρ but does not account for the accumulation of vehicles over time in a real roundabout scenario. Therefore, the subsequent analysis will focus on the evolution of the outflow number to better understand how traffic dynamics change over time within the roundabout system.

### 3.2. The Relationship Between Traffic Flow and Time

**Figure 9:**
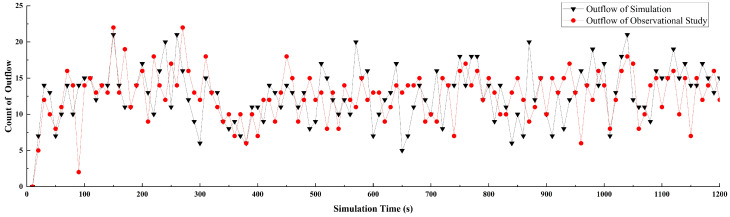
Vehicle count exiting the roundabout at fixed intervals.

**Figure 10:**
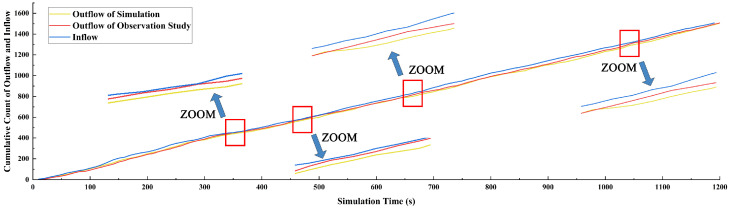
Cumulative count of outflow and inflow.

We present the temporal evolution of data on vehicles exiting the roundabout in both the empirical research and simulation, where data were recorded at 10 s intervals (Figure 9). It can be observed that the changes in the number of vehicles within the observation area are remarkably similar under the same input conditions. The peaks of both curves are identical and occur at almost the same time. Notably, anomalies are observed at 550, 650, and 870 s, but these quickly revert to normal values. To further validate the model’s simulation capability and assess the impact of these anomalies on the overall model, we compiled statistics on the number of vehicles exiting the simulated roundabout every 10 s, as well as the number of vehicles entering and exiting in the empirical research. These data were then accumulated and analyzed (Figure 10).

Simultaneously, we quantitatively compared the statistical correlation between the cumulative traffic flow data from empirical research and simulation. First, we conducted the Pearson test (Table 2) and concluded that the two datasets were statistically correlated (*p* < 0.05) and exhibited a strong positive correlation (Pearson correlation ≈ 1). Next, we examined the trend of the data using linear regression. Under the same observation time conditions, we used the cumulative vehicle count within the empirical research measurement area as the independent variable (i.e., *y* in Equation (5)) and the cumulative vehicle count from the simulation as the dependent variable (i.e., *x* in Equation (5)). The resulting equation is represented as follows:

(5) y=1.0142x−4.8854

**Figure 11:**
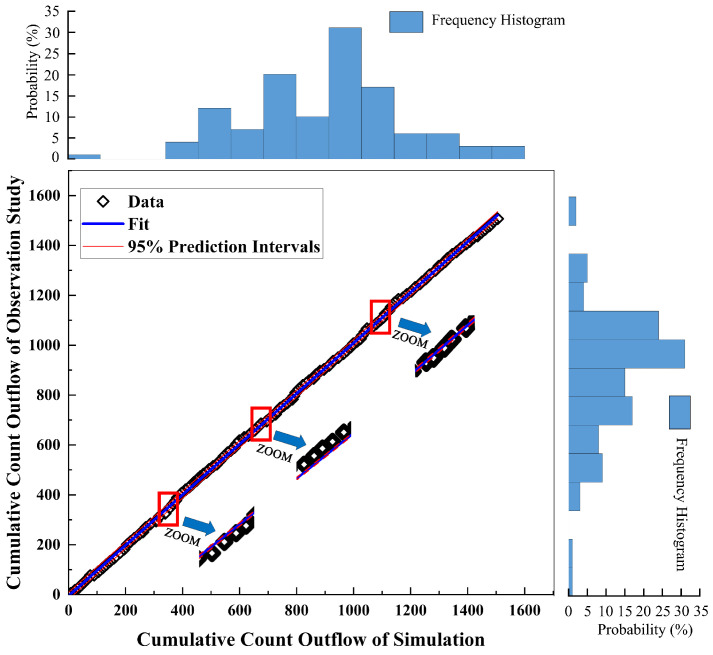
Linear regression of the cumulative outflow.

From the intuitive results in Figure 11, we can observe that the growth trends of each dataset are approximately equal. Based on the simulation and empirical study, under the input conditions of the empirical study, the model demonstrates relatively good matching.

**Figure 12:**
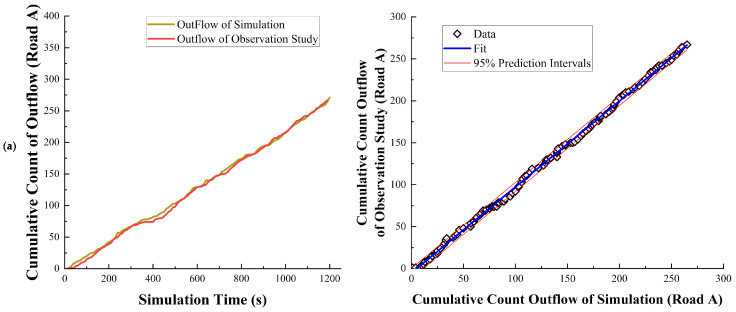

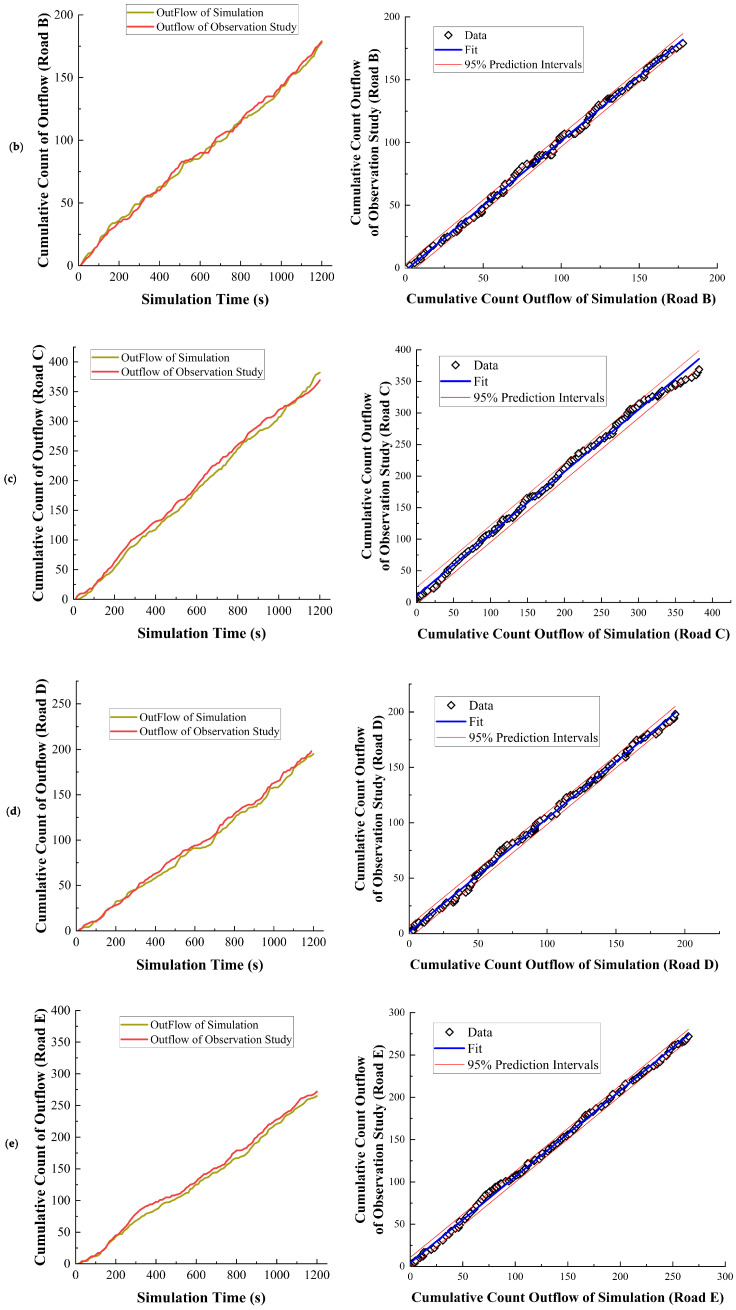

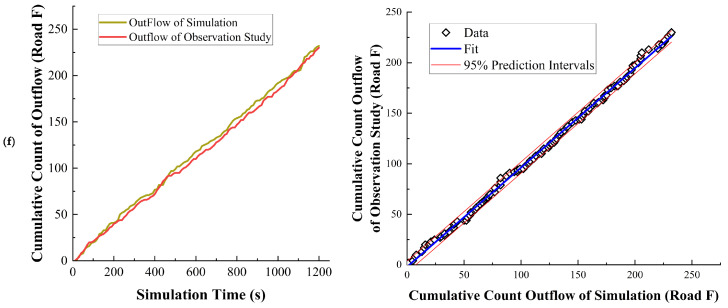
Comparison of cumulative and regression analysis. Specifically, (**a**–**f**) represent Roads A–F.

The data represented in Figure 9, Figure 10 and Figure 11 primarily focus on the overall relationship of vehicles on the roundabout. However, for a highly complex and large roundabout, the individual lane traffic flow better reflects real-world driving conditions. Therefore, a comparative analysis of the relationship between the traffic flow in each lane and time was conducted, along with Pearson correlation tests (Table 3) and regression analysis (Figure 12). Based on the analysis results, it is evident that each lane is highly correlated with the data from empirical research, and the regression analysis shows a very high level of matching. This indicates that the model exhibits excellent simulation capability and a high degree of correspondence with empirical research data for individual lanes of the roundabout.

### 3.3. Simulation Results’ Analysis

In this section, we will explore the issues identified in the roundabout through simulation results and analyze their causes. To this end, we conducted an analysis focusing on two aspects: (1) interactions between vehicles, and (2) the delay time incurred by each cell.

#### 3.3.1. Interaction Situation

**Figure 13:**
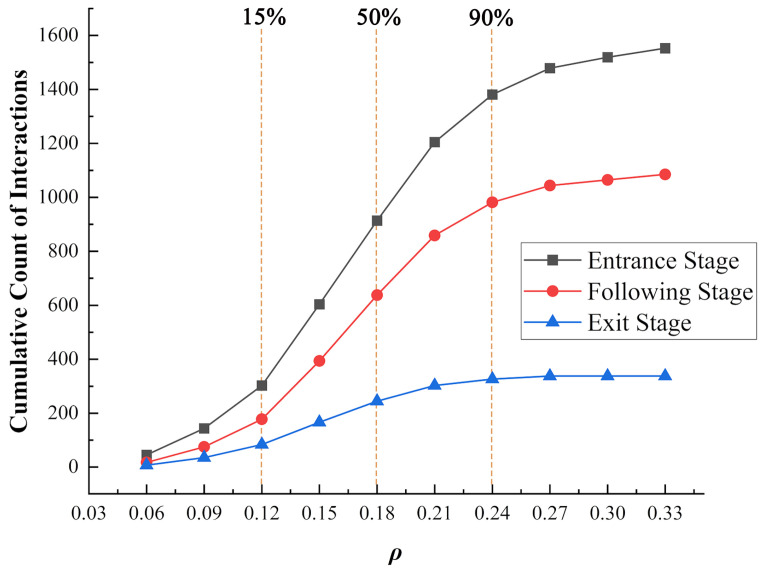
Cumulative count of interactions at different stages.

To gain a clearer understanding of the complex interaction environment encountered by vehicles on the roundabout, we statistically analyzed the stage at which each vehicle interacted and the number of surrounding vehicles involved in each interaction. Specifically, when a vehicle begins a lane-changing behavior, the system will identify its current stage and count the other vehicles within four cells in front and behind of the vehicle, both in the current lane and in the adjacent lane to which it is preparing to move (i.e., a 2 × 10 matrix), at the time of the update. Subsequently, the number of interacting vehicles at each of the three stages was plotted according to ρ at the time of interaction (Figure 13). It is evident that vehicles encounter the most interactions during the entrance stage, followed by the following stage, and then the exit stage. During the exit stage, most vehicles have already maneuvered into favorable positions through lane changes, reducing the probability of interactions with other vehicles. The majority of vehicle interactions occurred when in the range of 0.12 to 0.24 of ρ. This provides a reference for the ρ-based optimization strategy we propose later.

#### 3.3.2. Congested Areas Within the Roundabout

**Figure 14:**
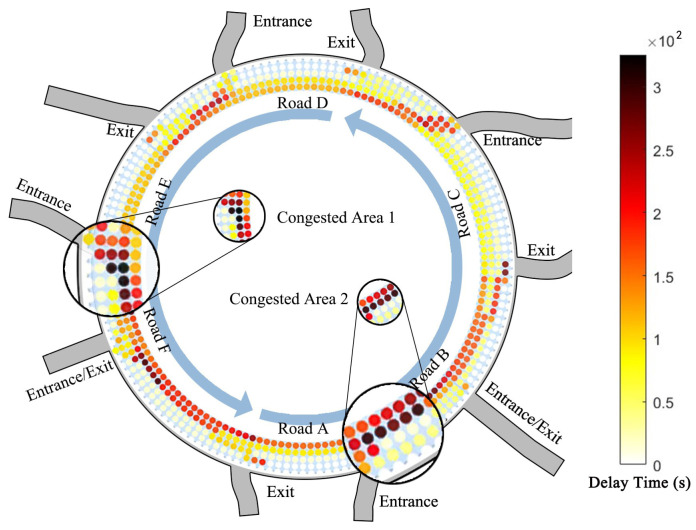
Roundabout congestion heatmap.

We conducted a statistical analysis of delay times for each cell in the simulation, which records the time in seconds after a vehicle remains stationary in a cell for two consecutive updates, continuing until the vehicle moves. The delay time recorded for each cell accumulates until the end of the simulation. We assigned the accumulated delay times for each cell to a 6 × 155 matrix on the basis of their positions (the data for the sixth row were too sparse and were therefore omitted from the figure) and then converted this matrix into a circular matrix via polar coordinates in MATLAB R2023b. The color of each cell reflects the delay time, with darker colors indicating longer delays (Figure 14). Through comparison, we found two areas of severe and frequent congestion: the area between the entrances of Road E and the exit of Road F, and the area between the entrances of Road A and the exit of Road B, which is consistent with the observed situation. Two factors collectively affect the traffic flow on the roundabout: (i) the proximity between the exits and entrances of adjacent roads is too close, leaving insufficient space for vehicles to change lanes (e.g., the congested areas 1 and 2 in Figure 14), and (ii) most vehicles choose to travel in R1-3, with R4 being used very infrequently (e.g., the congested area 1 in Figure 14). Additionally, for Road B, where vehicles enter the roundabout, the presence of a traffic island compresses the traffic space, resulting in a reduced traffic capacity.

Based on the analysis of these two sections, we can identify several key traffic flow characteristics that can help guide the optimization strategy: (1) vehicle interactions are particularly dense at the entry stage of the roundabout, emphasizing the need to reduce these interactions to prevent excessive congestion; (2) the optimization strategy should aim to effectively manage vehicle flow in high ρ areas to mitigate overall traffic delays; (3) special attention should be given to minimizing delays resulting from high-frequency interactions, particularly at critical points such as lane changes and merges.

## 4. Management and Optimization Strategies

In this section, we propose four targeted optimization strategies to address the issues identified through the analysis of results and empirical observations. Notably, vehicle sensors are integrated with roundabout infrastructure to enable real-time data exchange, thereby optimizing synchronization between vehicles and their surrounding environment. These advanced sensing technologies guide vehicles in optimized strategies, ultimately enhancing traffic efficiency and safety during the navigation process. Consequently, we explore strategies aimed at improving traffic efficiency, reducing delay times, and decreasing the frequency of dangerous interactions across different scenarios: (1) traffic efficiency—on the basis of the fundamental diagram, we demonstrate changes in roundabout efficiency when comparing the results of initial simulations with those of simulations implementing optimization strategies; (2) delay time—we compile the delay times to obtain a cumulative delay time graph for the entire roundabout; and (3) dangerous interactions—any involving more than two vehicles during a single interaction are considered relatively dangerous, indicating close proximity to surrounding vehicles and dense traffic; we record ρ and the frequency of these potentially hazardous interactions to assess the impact of optimization strategies on roundabout safety. Through these three metrics, we evaluate the improvement to the roundabout traffic brought by the optimization strategies.

### 4.1. Entrance Facility Optimization Strategy (Simulation 1)

Firstly, we note that the lane selection behavior of vehicles when entering the roundabout greatly influences both local and overall traffic efficiency. The results are as shown in the heatmap (Figure 14). Due to the improper configuration of traffic space for each lane entering the roundabout, the utilization rate of the outermost lane for right turns is very low. The low utilization rate of the outermost lane for right turns results in most vehicles interacting in the inner three lanes, leading to increased delay times and compromised safety. Therefore, we propose several rules:

(a) All vehicles aiming for the adjacent right road are prioritized to exit the roundabout on the outermost lane based on the updated rules.
(b) Vehicles traveling in R3 and aiming for the adjacent right road are allowed to change lanes outward at any time.

**Figure 15:**
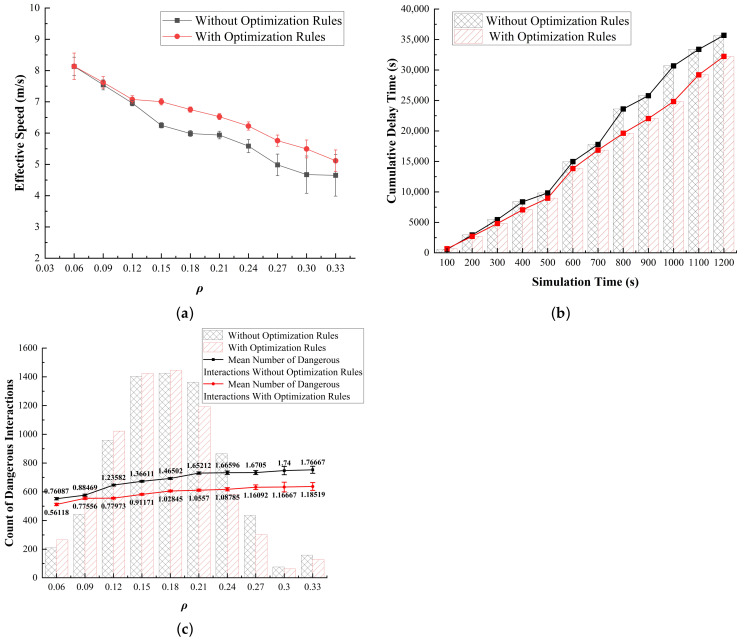
Results of Simulation 1. (**a**) Traffic efficiency; (**b**) delay time; (**c**) dangerous interactions.

It can be observed that as ρ increases, Simulation 1 exhibits a clear advantage in optimizing the effective speed on the roundabout (Figure 15a). Regarding delay time, after 700 s, the initial model experiences a rapid increase in delay time, while Simulation 1 successfully prevents this occurrence (Figure 15b). In terms of interaction frequency, a notable difference occurs after ρ exceeds 0.12. While the frequency of dangerous interactions among vehicles increases beyond this point, it gradually levels off (Figure 15c).

On the basis of the analysis of empirical research and the original model, we find a correlation with the situations indicated (Figure 15), which helps explain this phenomenon. As ρ increases, more vehicles entering the roundabout need to be considered, increasing the complexity of the traffic environment This intensification of ρ at entry points heightens the likelihood of interactions, particularly in the inner lanes, where vehicles may encounter greater competition for space and maneuverability. Consequently, the inner lanes experience higher interaction frequencies, which not only slow the overall traffic flow but also elevate the risk of unsafe maneuvers, as vehicles attempt to enter, merge, and change lanes within confined spaces.

Prioritizing the outermost lane for right-turning or exiting vehicles offers significant advantages, providing two primary benefits: (1) Prioritizing the outermost lane for right-turning vehicles enhances predictability, as drivers can anticipate that outer lanes will be primarily occupied by vehicles exiting, while inner lanes remain more reliable for longer-distance travel. (2) Allocating the outer lane for right turns reduces the pressure on inner lanes by minimizing lane changes, thus decreasing conflicts, enabling smoother trajectories, and preventing delays from spreading across the roundabout.

### 4.2. Road B Traffic Island Optimization Strategy (Simulation 2)

Here, our focus shifts to the traffic island at Road B within the scope of this study. Due to changes in urban land use functions, the traffic flow at Road B, which originally had bidirectional access to the roundabout, now only comprises vehicles exiting the roundabout as of the observation period. Additionally, the configuration of the traffic island does not align with the current traffic flow conditions, failing to effectively divert traffic and instead occupying the traffic space within the roundabout that was originally meant for normal vehicle flow. Considering congestion and real-world conditions, we modified the model map of the roundabout (the red traffic island shown in Figure 1b).

**Figure 16:**
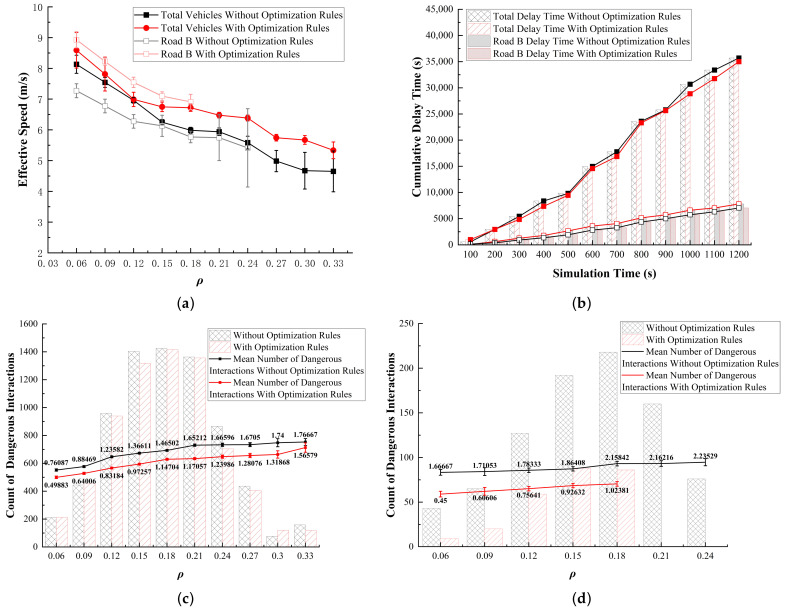
Results of Simulation 2, for Road B and all areas. (**a**) Traffic efficiency; (**b**) delay time; (**c**) dangerous interactions (all areas); (**d**) dangerous interactions (Road B).

**Figure 17:**
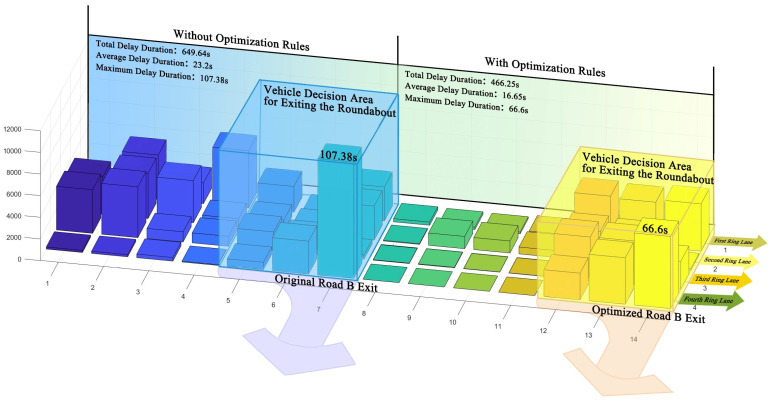
Comparison of congestion levels before and after the optimization at Road B.

The spatial optimization was conducted specifically for Road B. While analyzing the global data, the data for Road B were taken into account, specifically within the range from Road B to the nearest entrance, as no vehicles are entering the roundabout from Road B, and congestion only occurs at the traffic island in front of Road B. Therefore, only the situation where vehicles exit from Road B was considered. In the graph, the dark lines represent the global data, while the light lines represent the data for Road B. Through simulation optimization, new discoveries emerged in the fundamental diagram. We can observe that in the original simulation, the effective speed at Road B is lower compared to the global average. This further indicates the negative impact of the traffic island on the traffic efficiency at Road B (Figure 16a). For the delay time, although there is some improvement, the optimization effect remains unsatisfactory (Figure 16b). To further evaluate the impact of optimization on traffic efficiency, we performed a statistical analysis of cell delay times at the Road B exit (the area shown in Figure 17) in Simulation 2. Based on these values, we created a 3D heatmap. After calculating the delay time for this entrance area, it was found that the average delay time decreased by 28.23%, with the maximum value decreasing by 37.98%. In addition, in terms of the frequency of hazardous interactions, the occurrences at Road B were effectively controlled. This indicates that optimizing the traffic island at Road B can greatly improve both the efficiency and safety of traffic flow (Figure 16c). This suggests that optimizing the roundabout space has a direct positive impact on vehicle traffic efficiency.

Overall, the removal of the traffic island at Road B expands the available space for vehicle movement, significantly enhancing traffic flow efficiency by reducing interactions between vehicles. This improvement can be attributed to several pivotal factors. Primarily, the elimination of the traffic island facilitates a more streamlined vehicular trajectory, mitigating potential obstructions and delays associated with traffic infrastructure. By liberating the roadway from the constraints imposed by the traffic island, vehicles can traverse the area with increased velocity, thereby amplifying throughput capacity.

Conversely, the substantial reduction in delay times observed in the localized area (Road B) does not necessarily correlate with a comparable improvement in overall congestion metrics. This discrepancy can be ascribed to the intricate nature of the traffic system, wherein the dynamics of flow at the roundabout are influenced not only by the conditions at Road B but also by the configuration and operational efficiency of surrounding routes, traffic patterns, and signal management strategies. While the optimization measures implemented at Road B have yielded positive results, the alleviation of global delay is contingent upon addressing potential bottlenecks at other access points. Thus, achieving comprehensive enhancements in traffic efficiency necessitates a holistic approach that encompasses the dynamic interactions within the entire transportation network.

### 4.3. Threshold Control Strategy (Simulation 3)

It can be determined that the majority of vehicles in the roundabout operate within the ρ range of 0.12–0.24 vehicles/m^2^ (Figure 13, Figure 14, Figure 15 and Figure 16). At a ρ of about 0.12, there is a critical turning point for the traffic efficiency and safety of the roundabout. Effective speed exhibits a stable decline within the ρ range of 0.12–0.24, whereas after 0.24, it rapidly decreases. This indicates that the roundabout or specific local areas are approaching a bottleneck or gridlock. The focus of this study is precisely on reducing the frequency of such occurrences. Therefore, we propose the following optimization rules based on these observations:

(a) When ρ exceeds 0.24, vehicle entry to the roundabout is prohibited.
(b) For ρ between 0.18 and 0.24, the entry rate at each entrance is reduced by half, to 1.5 s.
(c) When ρ is below 0.18, vehicles enter the roundabout without restrictions.

**Figure 18:**
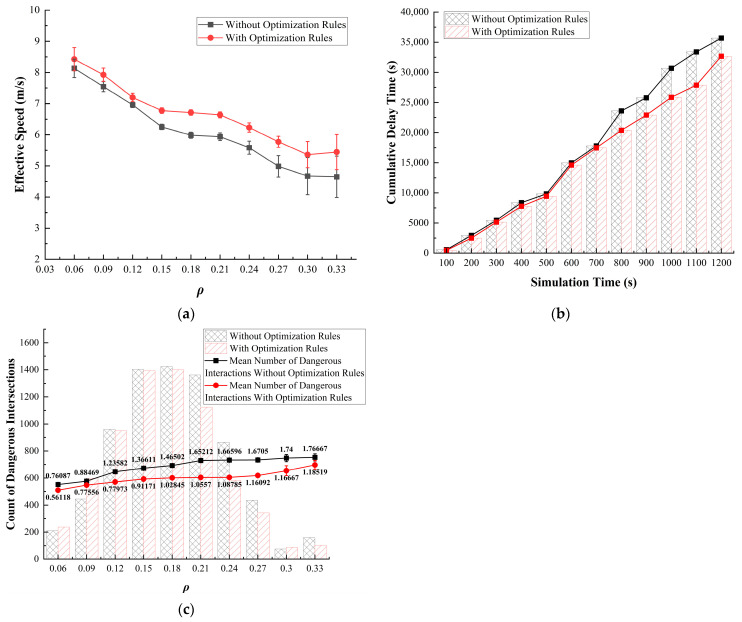
Results of Simulation 3. (**a**) Traffic efficiency; (**b**) delay time; (**c**) dangerous interactions.

When comparing the data before and after optimization, we can visually observe a significant positive impact on effective speed and dangerous interaction frequency at different ρ values (Figure 18a,c). For the delay time, similar to Strategy 1, there is a notable improvement in optimization after 700 s (Figure 18b). The findings suggest that even without implementing spatial optimization adjustments or prioritizing right-turn strategies, controlling the overall ρ can still yield meaningful improvements in both traffic efficiency and safety within the roundabout. By strategically regulating vehicle entry based on real-time ρ thresholds, this approach effectively maintains traffic flow within an optimal range, preventing conditions that could lead to excessive congestion or gridlock.

Compared to more aggressive spatial or directional optimizations, ρ control offers a more gradual and balanced enhancement of traffic dynamics. Rather than focusing on specific lanes or movement patterns, this strategy addresses the overall traffic flow, creating a smoother, more comfortable driving experience with fewer sudden bottlenecks. By keeping ρ at manageable levels, it minimizes abrupt changes in speed and reduces the likelihood of high-risk interactions, thereby enhancing the overall journey comfort and continuity for drivers.

### 4.4. Path Selection Based on Road Occupancy Rate Recognition Strategy (Simulation 4)

Now, we shift the focus to driver behavior. In this optimization phase, we will install signal signs on the roundabout and deploy ρ detectors. Through these signal detectors and guidance, drivers will be prompted to make informed lane selection decisions before entering or navigating the roundabout, thereby avoiding congestion and hazardous interactions. We optimize the decision-making algorithm in the original model, allowing drivers to choose the optimal path based on lane ρ when permitted by the algorithm. Based on ρ in the typical zone (Figure 7), the probability $P_{l}$ is calculated of vehicles for the three lanes using the following formula:

(6) $$P_{l}=\frac{1+\varepsilon \cdot \left(B_{l}/\sum_{l=1}^{3}B_{l}\right)}{\sum_{l=1}^{3}\left(1+\varepsilon \cdot \left(B_{l}/\sum_{l=1}^{3}B_{l}\right)\right)},l\in \left\{1,2,3\right\}$$

where $P_{l}$ represents the probability of selecting a lane in the typical zone, l=1,2,3 correspond to the inner, typical, and outer lanes, respectively, ε is a calibration factor that adjusts the vehicle’s sensitivity to lane ρ, with higher values placing a greater emphasis on lanes with lower ρ, and in this study, ε=1. The attractiveness of each lane, $B_{l}$, is defined by the maximum ρ $B_{\max}^{l}$ and the ρ of the current lane and lane l as follows:

(7) $$B_{l}=\max(0,(\rho_{\max}^{l}-\rho_{C}^{l})$$

where $\rho_{\max}^{l}$ and $\rho_{C}^{l}$ are the current ρ and the maximum ρ (for ease of calculation, the maximum ρ is 0.24) of each lane, respectively. $\rho_{C}^{l}$ is calculated based on lane l (Equation (3)).

Based on the probability $P_{l}$, the vehicle lane selection method can reasonably choose a path, but it is not a universal solution, so the following rules must be followed:

(a) The vehicle’s behavior is based on the original model.
(b) During the entrance stage, if a vehicle enters a congested lane, it will opportunistically switch to the inner lane (closer to the center).
(c) During the following stage, except for the innermost lane (R1), all vehicles consider changing lanes to the outer side when faced with congestion. If the vehicle in R1 has not yet reached the advanced lane-changing cell (the green block in Figure 5), it will continue changing lanes to avoid congestion.

**Figure 19:**
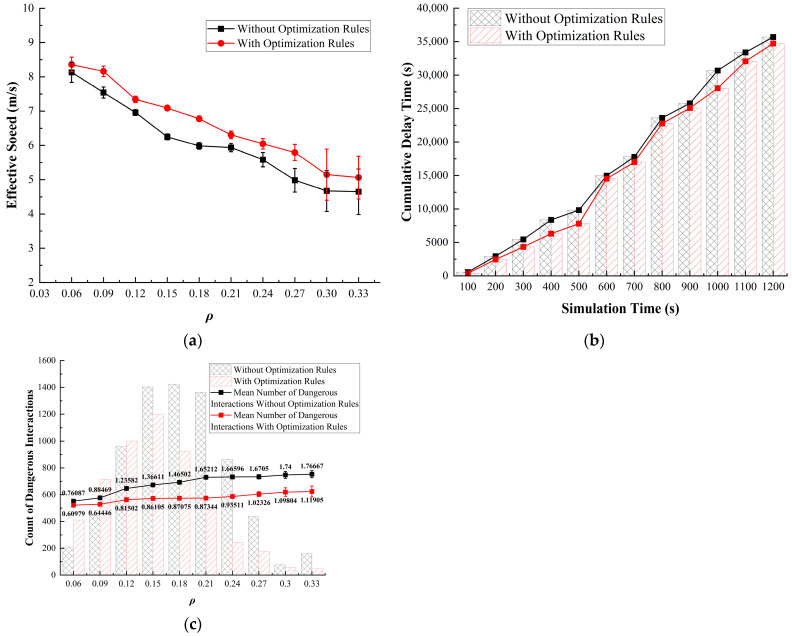
Results of Simulation 3. (**a**) Traffic efficiency; (**b**) delay time; (**c**) dangerous interactions.

We found significant reductions in both the fundamental diagram and frequency of dangerous interactions (Figure 19a,c). However, the positive impact on delay time was relatively weak (Figure 19b). This result highlights a key limitation in optimizing roundabout traffic flow solely through driver decision-making improvements: once congestion reaches a certain threshold within the roundabout, even rational, optimized lane choices by drivers can only mitigate the adverse effects to a limited extent. This is because, in highly congested environments, the physical constraints of the roundabout—such as limited lane space, high ρ, and reduced maneuverability—create a situation where incremental improvements in lane selection cannot fully overcome the inherent bottlenecks.

In such conditions, drivers have limited opportunities to alter their position or change lanes effectively, as the choices become constrained by the proximity and density of other vehicles. Optimizing driver behavior can reduce immediate issues such as sudden deceleration or frequent lane changing, which may improve flow continuity and reduce the occurrence of risky interactions. In fact, the scope for “rational decisions” shrinks as the congestion level rises.

Overall, the effectiveness of decision-based optimizations relies heavily on pre-emptive measures—drivers need adequate space and time to adjust their positions based on congestion predictions or lane densities. When the roundabout is already congested, these proactive decisions become reactive, leading to only minor adjustments in an already overloaded system. In such scenarios, interventions beyond driver decision-making, such as physical modifications (Simulations 1–2) or dynamic entry restrictions based on real-time ρ (Simulation 3), are necessary to achieve meaningful improvements in traffic flow and delay reduction.

Therefore, combining optimization strategies from different perspectives can further enhance the overall traffic efficiency of the roundabout.

## 5. Discussion and Conclusions

To effectively increase roundabout traffic capacity, optimizing vehicle flow is an important field of research, facilitated by advanced driving behavior sensors as well as vehicle-based and cloud–vehicle communication technologies. In this context, onboard vehicle sensors are pivotal in effectively implementing and comprehensively analyzing optimization measures, particularly by guiding driving decisions, enabling real-time sensing, and facilitating seamless data exchange. However, a more foundational prerequisite lies in the development of precise traffic flow models and effective optimization strategies.

Recognizing this need, we propose a Three-Stage Cellular Automata (TSCA) model for large roundabouts, which is based on a Cellular Automata framework and calibrated via empirical observation data. When comparing the simulation results with actual observations, we found that the TSCA model accurately reproduces the complex traffic conditions in large roundabouts. Therefore, we propose targeted optimization strategies based on the issues identified in both observations and simulations. By comparing the improvements in traffic efficiency, delay time, and frequency of dangerous interactions, we analyzed the impacts of the four optimization strategies in enhancing roundabout traffic quality. After organizing the data, we found that the three comparison indicators exhibit a strong correlation with changes in ρ, with most vehicles operating within the ρ range of 0.12 to 0.24. To further compare the improvement effects of different optimization strategies, we compiled the average speed, total delay time, total number of dangerous interactions, and frequency of vehicles (Table 4).

It can be observed that all four optimization strategies have a positive effect. Simulation 4 demonstrated the best overall performance. Specifically, traffic efficiency, delay time, dangerous interactions, and dangerous interaction frequency improved by 15.65%, 6.50%, 28.32%, and 38.03%, respectively. Although Simulation 1 showed limited improvements in traffic efficiency, it achieved the greatest reduction in delay time, with a 6.90% decrease (Table 4). Additionally, the average speed in the optimized section increased to 7.74 m/s, representing an improvement of 25.04%. The other strategies produced results between these two extremes, each demonstrating its own strengths and weaknesses. Overall, vehicle behavior-based optimizations (Simulations 3 and 4) showed superior results for overall roundabout traffic quality compared to space-based strategies. This highlights the integrated nature of the roundabout system, where improving driver decision-making has a greater impact than isolated spatial changes. While space-based optimizations can ease congestion in specific areas, they do not address the interconnected dynamics across the roundabout. Behavioral optimizations offer a flexible approach by directly influencing drivers’ responses to real-time conditions, guiding them toward safer, more efficient behaviors such as choosing lanes based on density and adjusting speed to reduce risky interactions. This adaptability is especially beneficial in the roundabout’s dynamic environment, where localized congestion can impact the entire system. Therefore, combining optimization strategies from different perspectives can further enhance the overall traffic efficiency of the roundabout.

In recent years, the integration of ITS into traffic management, particularly through autonomous vehicles and advanced onboard sensors, has garnered significant attention as a potential solution to traffic-related challenges. Ma et al. [41] provided a comprehensive review of advancements in intersection control, highlighting the potential of ITS to reduce accidents and improve traffic flow, especially in environments where traditional vehicles coexist with connected and autonomous vehicles. Building on the advancements in ITS for traffic flow, research has also explored the impact of autonomous and connected vehicles in mixed traffic environments, as demonstrated by Zhu et al. [42]. Onboard vehicle sensors are critical for autonomous vehicles to accurately perceive their surroundings. In complex traffic scenarios like roundabouts, autonomous vehicles rely on the integration of multiple sensors—such as cameras, radar, and LiDAR—to maintain precise environmental awareness. Wang et al. [43] emphasizes the importance of multi-sensor fusion in real-time data processing, enabling autonomous systems to make informed and safe decisions in dynamic traffic environments. This emphasis on multi-sensor fusion underscores the increasing significance of onboard vehicle sensors, which are pivotal for advancing both present optimization strategies and future research frameworks, such as the one proposed in this study. One of the advantages of this study is that it utilizes onboard vehicle sensors to deploy optimization strategies and further prepares the framework for the future integration of autonomous vehicles.

Overall, as a crucial component of urban transportation systems, large roundabouts serve as vital hubs, ensuring the efficient movement and coordination of traffic. Therefore, optimizing traffic flow within large roundabouts has become a pressing topic that demands in-depth research. With the advancement of onboard vehicle sensors and other cutting-edge technologies, ITSs now offer an effective means to enhance both the efficiency and stability of traffic flow. Specifically, onboard vehicle sensors within ITSs play a pivotal role in improving roundabout traffic performance by guiding driving behavior, facilitating real-time data collection and interaction, and enhancing the system’s overall effectiveness, particularly in terms of reducing energy consumption, controlling emissions, predicting traffic patterns, and optimizing flow. Given these advancements, traffic flow models and optimization strategies that incorporate onboard vehicle sensors further refine the application of ITSs in roundabout traffic research, paving the way for more scientifically grounded and efficient traffic management.

In light of this, our study, based on empirical observations, proposes the TSCA model to simulate the complex traffic flow characteristics in large roundabouts. The innovations of this study lie first in the development of a novel model designed to simulate complex traffic flow on large roundabouts, achieving favorable simulation results. Secondly, based on identified issues within the roundabout, targeted optimization strategies were proposed to enhance various aspects of roundabout performance, including traffic efficiency, comfort, and safety. This study holds significant practical value, particularly with the support of advanced onboard vehicle sensors, which not only facilitate the real-world implementation of our proposed optimization strategies but also effectively detect non-compliance and provide real-time warnings to help drivers adhere to prescribed traffic management strategies.

When applied to large roundabouts with multiple entrances and lanes, the TSCA model offers an effective and versatile solution, provided that these key principles—drawn from both empirical research and practical applications—are followed. This strong alignment with empirical results underscores the robustness of the model. The model aims to analyze the factors that enhance roundabout traffic capacity by comparing various optimization strategies, with key indicators such as traffic efficiency, delay time, and both the number and frequency of dangerous interactions. The results clearly show that the model effectively replicates the complex traffic flow in large roundabouts, and the four proposed optimization strategies all improve the roundabout traffic capacity to varying degrees. Although somewhat heuristic, these optimization strategies have been shown to help identify guiding principles for roundabout management and spatial design. Furthermore, these strategies may, to some extent, address the current lack of standardized rules for managing roundabout traffic and have been numerically validated for their performance. However, field validation will be required before large-scale implementation to fully determine the potential of these optimization strategies.

Finally, we would like to discuss some limitations and anticipated improvements of this work. First, owing to constraints in video recording, data could only be manually collected, and detailed vehicle trajectories were unavailable. Second, there are still limitations in the micro-level study of vehicle traffic behavior on a roundabout using the TSCA model, as the positions of vehicles on the roundabout also affect decision-making to some extent. Third, this study only considered interactions between motor vehicles on the roundabout; however, in reality, interactions between motor vehicles and nonmotorized vehicles are not uncommon, and even interactions with pedestrians (although rare) occasionally occur. The TSCA model overlooks the heterogeneity of interaction objects because this study focuses on the decision-making behavior of vehicles in complex environments, aiming to investigate the influence of imagined path distance on lane selection decisions and the optimization of roundabout spatial design and related strategies. Finally, although the management and guidance strategies we proposed were tested with the tools painstakingly validated in our research scenario, empirical validation should be conducted before these strategies are widely implemented in practice to consolidate their effectiveness and check for any overlooked side effects. These are natural subsequent steps following the initial work.

## Figures and Tables

**Table 1:** Statistical data of the relationship between ΔR and lane choice behavior (for ΔR=1, 2, 3).

	ΔR = 1			ΔR = 2				ΔR = 3			
Entrance Road	Normal Count	Exception Count	Selected Lane	Normal Count	Exception Count	Selected Lane		Normal Count	Exception Count	Selected Lane	
			R2			R3	R1			R3	R2
Road A	20→R3&R4	1	1→R2	20→R2	7	7→R3	-	5→R1	2	-	2→R2
Road B	-	-	-	-	-	-	-	-	-	-	-
Road C	28→R3&R4	-	-	14→R2	3	-	3→R1	20→R1	11	-	11→R2
Road D	14→R3&R4	-	-	18→R2	1	1→R3	-	5→R1	3	-	3→R2
Road E	12→R3&R4	-	-	25→R2	10	7→R3	3→R1	1→R1	6	1→R3	5→R2
Road F	9→R3&R4	1	1→R2	5→R2	-	-	-	10→R1	12	-	12→R2

**Table 2:** Statistical data of the relationship between ΔR and lane choice behavior (for ΔR=4, 5, 6).

	ΔR = 4			ΔR = 5			ΔR = 6		
Entrance Road	Normal Count	Exception Count	Selected Lane	Normal Count	Exception Count	Selected Lane	Normal Count	Exception Count	Selected Lane
			R2			R2			-
Road A	27→R1	5	5→R2	7→R1	1	1→R2	2→R1	-	-
Road B	-	-	-	-	-	-	-	-	-
Road C	13→R1	3	3→R2	3→R1	4	4→R2	8→R1	-	-
Road D	3→R1	1	1→R2	22→R1	-	-	-	-	-
Road E	16→R1	9	9→R2	8→R1	2	2→R2	1→R1	-	-
Road F	4→R1	1	1→R2	2→R1	-	-	-	-	-

**Table 3:** The Pearson test results.

Data Type	Number of Data Pairs	*p*-Value	Pearson Correlation
Aggregate	120	0.000	0.999876767
Road A	120	0.000	0.999400183
Road B	120	0.000	0.998861147
Road C	120	0.000	0.998166275
Road D	120	0.000	0.998830072
Road E	120	0.000	0.999191377
Road F	120	0.000	0.999070709

**Table 4:** Comparison of optimization simulation models for key traffic indicators.

	Average Speed (m/s)	Delay Time (s)	Count of Dangerous Interactions	Frequency of Dangerous Interactions
Original Model	6.07	209,076	7340	1.42
Simulation 1	6.60	182,856	7052	0.97
Simulation 2	6.63	201,614	7048	1.07
Simulation 3	6.64	187,047	6823	0.97
Simulation 4	7.02	195,481	5261	0.88

## Data Availability

Data available on request due to privacy restrictions.

## Appendix A

### Appendix A.1. Probability of Vehicles Choosing Exit Roads

1. When the initial road is Road A:
  (1) The probability of a fast car appearing is 67.7%.
  (2) The probabilities of choosing Road A, Road B, Road C, Road D, Road E, and Road F as the aim roads are 2.1%, 22.6%, 27.8%, 7.2%, 33.0%, and 8.2%, respectively.
  (3) When these roads are the aim roads, the probabilities of vehicles entering the roundabout from the right lane of Road A are 5.0%, 81.0%, 0%, 0%, 36.3%, and 0%.
2. No vehicles exit from Road B; hence, the probabilities are all 0.
3. When the initial road is Road C:
  (1) The probability of a fast car appearing is 61.8%.
  (2) The probabilities of choosing Road A, Road B, Road C, Road D, Road E, and Road F as the aim roads are 15.0%, 6.5%, 7.5%, 26.2%, 15.9%, and 29.0%, respectively.
  (3) When these roads are the aim roads, the probabilities of vehicles entering the roundabout from the right lane of Road C are 0%, 0%, 0%, 90.9%, 76.9%, and 100.0%.
4. When the initial road is Road D:
  (1) The probability of a fast car appearing is 68.8%.
  (2) The probabilities of choosing Road A, Road B, Road C, Road D, Road E, and Road F as the aim roads are 11.9%, 6.0%, 32.8%, 0%, 20.9%, and 28.4%, respectively.
  (3) When these roads are the aim roads, the probabilities of vehicles entering the roundabout from the right lane of Road D are 100.0%, 100.0%, 60.0%, 50.0%, 100.0%, and 100.0%.
5. When the initial road is Road E:
  (1) The probability of a fast car appearing is 55.3%.
  (2) The probabilities of choosing Road A, Road B, Road C, Road D, Road E, and Road F as the aim roads are 38.9%, 7.8%, 27.8%, 11.1%, 1.1%, and 13.3%, respectively.
  (3) When these roads are the aim roads, the probabilities of vehicles entering the roundabout from the right lane of Road E are 75.0%, 54.5%, 55.5%, 50.0%, 50.0%, and 0%.
6. When the initial road is Road F:
  (1) The probability of a fast car appearing is 83.3%.
  (2) The probabilities of choosing Road A, Road B, Road C, Road D, Road E, and Road F as the aim roads are 22.7%, 11.4%, 50.0%, 11.4%, 4.5%, and 0%, respectively.
  (3) When these roads are the aim roads, the probabilities of vehicles entering the roundabout from the right lane of Road F are 100.0%, 100.0%, 100.0%, 100.0%, 100.0%, and 100.0%.

### Appendix A.2. Probability of Vehicles Choosing Target Lanes

Since no vehicles enter the roundabout from Road B, this scenario does not consider Road B.

(1) When the ΔR=1:

For all initial roads: the probability of choosing R3 and R4 as the target lane is 98.0%, with the choice between R3 and R4 being random; otherwise, R2 is chosen.

(2) When the ΔR=2:

For initial Road A: the probability of choosing R2 as the target lane is 74.0%; otherwise, R3 is chosen.

For initial Road C: the probability of choosing R2 as the target lane is 82.0%; otherwise, R1 is chosen.

For initial Road D: the probability of choosing R2 as the target lane is 95.0%; otherwise, R3 is chosen.

For initial Road E: the probability of choosing R2 as the target lane is 71.0%; the probabilities of choosing R3 and R1 are 20.0% and 9.0%, respectively.

For initial Road F: the probability of choosing R2 as the target lane is 100.0%.

(3) When the ΔR=3:

For initial Road A, Road C, Road D and Road F: the probabilities of choosing R1 as the target lane are 72.0%, 65.0%, 63.0%, and 46.0%, respectively; otherwise, R2 is chosen.

For initial Road E: the probability of choosing R2 as the target lane is 70.0%; the probabilities of choosing R3 and R1 are 16.0% and 14.0%, respectively.

(4) When the ΔR=4:

For initial Road A, Road C, Road D, Road E, and Road F: the probabilities of choosing R1 as the target lane are 85.0%, 82.0%, 75.0%, 64.0%, and 80.0%, respectively; otherwise, R2 is chosen.

(5) When the ΔR=5:

For initial Road A, Road C, Road D, Road E, and Road F: the probabilities of choosing R1 as the target lane are 80.0%, 43.0%, 100.0%, 80.0%, and 100.0%, respectively; otherwise, R2 is chosen.

(6) When the ΔR=6:

For all initial roads, only R1 is chosen as the target lane.

## References

[1] Alaba F.A., Oluwadare A., Sani U., Oriyomi A.A., Lucy A.O., Najeem O. (2024). Enabling Sustainable Transportation Through IoT and AIoT Innovations. Artificial Intelligence of Things for Achieving Sustainable Development Goals.

[2] New Infrastructure Is a Strategic Cornerstone to Support the Development of New Formats, *New Ind. New Serv.*. https://www.jfdaily.com/staticsg/res/html/web/newsDetail.html?id=720954&sid=300.

[3] Chen S., Hu X., Zhao J., Wang R., Qiao M. (2024). A Review of Decision-Making and Planning for Autonomous Vehicles in Intersection Environments. World Electr. Veh. J..

[4] Gorrini A., Crociani L., Vizzari G., Bandini S. (2018). Observation Results on Pedestrian-Vehicle Interactions at Non-Signalized Intersections Towards Simulation. Transp. Res. Part F Traffic Psychol. Behav..

[5] Chen L., Qiao C., Zhang J., Xie C.Z.T., Tang T.Q., Chen Y. (2024). Behavioral Patterns of Children During Emergency Evacuations: A Comparative Analysis of Experimental Observations and Simulation Results. J. Stat. Mech. Theory Exp..

[6] Wang F., Di Z., Zhang N., Ruan Y., Luo B., Wang Y., Liu X. (2024). Analysis of Construction Process and Configuration Automatic Monitoring for the Spoke-Type Single-Layer Cable Net Structure. Buildings.

[7] Wang L., Ding M., Ruan Y., Luo B., Guo J. (2023). Error Influence Simulation of the 500 m Aperture Spherical Radio Telescope Cable-Net Structure Based on Random Combinations. Sustainability.

[8] Fu L., Zhang Y., Qin H., Shi Q., Chen Q., Chen Y., Shi Y. (2023). A modified social force model for studying nonlinear dynamics of pedestrian-e-bike mixed flow at a signalized crosswalk. Chaos Solitons Fractals.

[9] Fu L., Zhang Y., Chen Q., He Y., Shen C., Shi Y. (2025). Behavioral characteristics of bidirectional pedestrian-e-bike mixed flow at a signalized crosswalk: An experimental study. Travel Behav. Soc..

[10] Ahmed A., Rizvi S.F.A., Ahmad F. (2024). Modelling Merging Behaviour of Drivers in Heterogeneous Traffic at Roundabouts. Eur. Transp./Trasp. Eur..

[11] Rossi R., Meneguzzer C., Orsini F., Gastaldi M. (2020). Gap-Acceptance Behavior at Roundabouts: Validation of a Driving Simulator Environment Using Field Observations. Transp. Res. Procedia..

[12] Silvano A.P., Ma X., Koutsopoulos H.N. (2015). When Do Drivers Yield to Cyclists at Unsignalized Roundabouts? Empirical Evidence and Behavioral Analysis. Transp. Res. Rec..

[13] Li C., Liu S., Xu G., Cen X. (2022). Influence of Driver’s Yielding Behavior on Pedestrian-Vehicle Conflicts at a Two-Lane Roundabout Using Fuzzy Cellular Automata. J. Cent. South Univ..

[14] Patnaik A.K., Rao S., Krishna Y., Bhuyan P.K. (2017). Empirical Capacity Model for Roundabouts Under Heterogeneous Traffic Flow Conditions. Transp. Lett..

[15] Yoshioka K., Hnakamura H., Shimokawa S., Morita H. (2017). An Analysis on the Impact of Roundabout Geometric Elements on Driving Behavior. J. East. Asia Soc. Transp. Stud..

[16] Guerrieri M., Mauro R., Parla G., Tollazzi T. (2018). Analysis of Kinematic Parameters and Driver Behavior at Turbo Roundabouts. J. Transp. Eng. Part A Syst..

[17] Zhou L., Zhang L., Liu C.S. (2022). Comparing Roundabouts and Signalized Intersections Through Multiple- Model Simulation. IEEE Trans. Intell. Transp. Syst..

[18] Lihejiang Roundabout Frequently Experiences Traffic Congestion During Peak Periods. Qinzhou City Government. http://zmqzcyyq.gxzf.gov.cn/gzhd/rdhf/t8100519.shtml.

[19] Cheng W., Zhu X., Song X. (2016). Research on Capacity Model for Large Signalized Roundabouts. Procedia Eng..

[20] Xie C.Z., Tang T.Q., Zhang B.T., Xiang H.J. (2022). Experiment, Model, and Simulation of Pedestrian Flow Around a Training School Classroom During the After-Class Period. Simulation.

[21] Wang R., Ruskin H.J. (2006). Modelling Traffic Flow at Multi-Lane Urban Roundabouts. Int. J. Mod. Phys. C.

[22] Bie J., Lo H.K., Wong S.C. (2010). Capacity Evaluation of Multi-Lane Traffic Roundabout. J. Adv. Transp..

[23] Tumminello M.L., Macioszek E., Granà A. (2024). Insights into Simulated Smart Mobility on Roundabouts: Achievements, Lessons Learned, and Steps Ahead. Sustainability.

[24] Gan J., Zhang J., Liu Y. (2024). Research on Behavioral Decision at an Unsignalized Roundabout for Automatic Driving Based on Proximal Policy Optimization Algorithm. Appl. Sci..

[25] Asia’s Largest Transportation Complex: Wuhan Optics Valley Plaza. https://www.163.com/dy/article/EO2SSR6D051480KF.html.

[26] The Traffic Volume of Guanggu Roundabout Is Overloaded During Peak Hours. https://hb.sina.com.cn/news/b/2015-04-09/detail-iavxeafs4882667.shtml.

[27] Harsono T., Arai K. (2024). Modeling Micro Traffic Flow Phenomena Based on Vehicle Types and Driver Characteristics Using Cellular Automata and Monte Carlo. Int. J. Adv. Comput. Sci. Appl..

[28] Qiao Y., Xue Y., Wang X., Cen B.-l., Wang Y., Pan W., Zhang Y.-x. (2021). Investigation of PM Emissions in Cellular Automata Model with Slow-to-Start Effect. Phys. A Stat. Mech. Its Appl..

[29] Xue W., Zhao Y., Jia J. Study on Characteristics of Multi-Speed Mixed Traffic Flow at a Single-Loop Roundabout. Proceedings of the International Conference on Frontiers of Electronics, Information and Computation Technologies.

[30] Ez-zahar A., Lakouari N., Oubram O., Aguilar J.G.V., Ez-zahraouy H. Simulation Analysis of Traffic Management in Roundabout Systems. Proceedings of the International Conference on Frontiers of Electronics, Information and Computation Technologies.

[31] Tang T.Q., Xie C.Z., Chen L. (2019). Modeling and Simulating the Pedestrian Flow in a Training School Classroom During the Pickup Period. Phys. A Stat. Mech. Its Appl..

[32] Marzoug R., Lakouari N., Pérez Cruz J.R., Vega Gómez C.J. (2022). Cellular Automata Model for Analysis and Optimization of Traffic Emission at Signalized Intersection. Sustainability.

[33] Wang X., Xue Y., Cen B., Zhang P., He H.D. (2020). Study on pollutant emissions of mixed traffic flow in cellular automaton. Phys. A Stat. Mech. Its Appl..

[34] Lakouari N., Oubram O., Bassam A., Hernandez S.E.P., Marzoug R., Ez-Zahraouy H. (2020). Modeling and simulation of CO_2_ emissions in roundabout intersection. J. Comput. Sci..

[35] Huang D. (2015). Modeling Gridlock at Roundabout. Comput. Phys. Commun..

[36] Dong R.H., Xue W.D., Zhu C.S. (2014). Study on the Mixing Traffic in a Single-Lane Roundabout with Open Boundary Condition. Adv. Mater. Res..

[37] Xie C.Z., Tang T.Q., Zhang B.T., Nicolas A. (2023). Adult–Child Pairs Walking Down Stairs: Empirical Analysis and Optimal-Step-Based Modeling of a Complex Pedestrian Flow, with an Exploration of Flow-Improvement Strategies. J. Stat. Mech. Theory Exp..

[38] Laarej A., Karakhi A., Khallouk A., Lakouari N., Ez-Zahraouy H. (2021). Dissipation Energy and Satisfaction Rate for a Two-Lane Traffic Model with Two Types of Vehicles. Chin. J. Phys..

[39] Wei L., Li W., Liang H., Luo F. (2022). Traffic Flow Characteristics of Speed Limited Roads Based on Cellular Automata NaSch Traffic Flow Model. The International Symposium on Computer Science, Digital Economy and Intelligent Systems.

[40] Regragui Y., Moussa N. (2018). A Cellular Automata Model for Urban Traffic with Multiple Roundabouts. Chin. J. Phys..

[41] Ma W., Li J., Yu C. (2023). Intersection Control in Mixed Traffic with Connected Automated Vehicles: A Review of Recent Developments and Research Frontiers. China J. Highw. Transp..

[42] Zhu H.B., Zhou Y.J., Wu W.J. (2020). Modeling Traffic Flow Mixed with Automated Vehicles Considering Drivers’ Character Differences. Phys. A Stat. Mech. Its Appl..

[43] Wang C., Storms K., Zhang N., Winner H. (2024). Runtime Unknown Unsafe Scenarios Identification for SOTIF of Autonomous Vehicles. Accid. Anal. Prev..

